# END-OF-LIFE CARE PLANNING AND SATISFACTION AMONG THE HEALTH AND RETIREMENT STUDY DECEDENTS

**DOI:** 10.1093/geroni/igac059.1398

**Published:** 2022-12-20

**Authors:** Zahra Rahemi, Ayse Malatyali, Tom Cidav, Olga Jarrín, Cheryl Dye, Christopher McMahan

**Affiliations:** Clemson University, Greenville, South Carolina, United States; University of Central Florida, Orlando, Florida, United States; Johns Hopkins University, Baltimore, Maryland, United States; Rutgers, The State University of New Jersey, New Brunswick, New Jersey, United States; Clemson University, Clemson, South Carolina, United States; Clemson University, Clemson, South Carolina, United States

## Abstract

The frequency and timing of advance care planning among individuals living with cognitive impairments vary by race/ethnicity and other sociodemographic factors. This study examined relationships between advance care planning and end-of-life care satisfaction among participants in the Health and Retirement Study (Exit files 2002-2018). Among decedents with cognitive impairment (n=3,102), Black and Hispanic participants were less likely to have a living will (OR=0.22, 0.19) and less likely to discuss end-of-life care (OR=0.422, 0.544) compared to White and non-Hispanic participants, respectively. Black and Hispanic participants were more likely to prefer all possible end-of-life care (OR=3.29, 3.34) and less likely to refuse extensive-care measures (OR=0.34, 0.48) compared to White and non-Hispanic participants. Participants dissatisfied with end-of-life care were 48% less likely to have a living will. End-of-life care planning disparities among racial/ethnic groups can inform interventional and educational programs to improve equity in end-of-life care.

